# *Encephalitozoon intestinalis* infection increases host cell mutation frequency

**DOI:** 10.1186/1750-9378-8-43

**Published:** 2013-11-04

**Authors:** Cory Ann Leonard, Maria Schell, Robert Vincent Schoborg, James Russell Hayman

**Affiliations:** 1 Department of Biomedical Sciences, James H. Quillen College of Medicine, East Tennessee State University, Johnson City, TN 37614-0579, USA

**Keywords:** *Encephalitozoon intestinalis*, Microsporidia infection, Microsporidiosis, Big Blue™ mouse, Mutation frequency, Opportunistic infections and cancer

## Abstract

**Background:**

Microsporidia are obligate intracellular opportunistic fungi that cause significant pathology in immunocompromised hosts. However, 11 percent of immunocompetent individuals in the general population are microsporidia-seropositive, indicating that severe immune suppression may not be a prerequisite for infection. *Encephalitozoon intestinalis* is transmitted in contaminated water and initially infects gastro-intestinal enterocytes, leading to diarrheal disease. This organism can also disseminate to many other organs. A recent report suggests that microsporidia can establish persistent infections, which anti-fungal treatment does not eradicate. Like other intracellular pathogens, microsporidia infection stresses the host cell and infected individuals have elevated hydrogen peroxide and free radical levels.

**Findings:**

As oxidative stress can lead to DNA damage, we hypothesized that *E. intestinalis-*infection would increase host cell nuclear mutation rate. Embryo fibroblasts from Big Blue^TM^ transgenic mice were *E. intestinalis*-infected and host nuclear mutation frequency was determined by selection of temperature-sensitive *c-II* gene mutant λ phage. The host mutation frequency in *E. intestinalis*-infected cultures was 2.5-fold higher than that observed in either mock-infected cells or cells infected with UV-inactivated *E. intestinalis* spores.

**Conclusions:**

These data provide the first evidence that microsporidia infection can directly increase host cellular mutation frequency. Additionally, some event in the microsporidia developmental cycle between host cell attachment and parasitophorous vacuole formation is required for the observed effect. As there is considerable evidence linking infection with other intracellular pathogens and cancer, future studies to dissect the mechanism by which *E. intestinalis* infection increases host mutation frequency are warranted.

## Findings

### Background

Microsporidia are intracellular parasitic fungi that infect many animals, including humans. Microsporidiosis in the immunocompromised first became evident during the Acquired Immunodeficiency Syndrome (AIDS) pandemic, but remains a concern in Human Immunodeficiency Virus (HIV)-infected individuals without access to therapy, as well as those immunosuppressed due to cancer therapy, diabetes, organ transplants, and autoimmune disease [1]. However, 11% of HIV-negative individuals in the general population are Microsporidia seropositive, suggesting that while overt disease may not be widespread, asymptomatic infections of healthy individuals are relatively common [2]. Kotkova *et al.* demonstrated that *Encephalitozoon cuniculi*-infected, albendazole-treated BALB/c mice can develop disseminated infection if they are immunosuppressed post-treatment [3]. As chronic microsporidia infections occur in humans [4], these data suggest such infections may be refractory to treatment [3]. Thus, microsporidia are important emerging pathogens and are classified by the US National Institutes of Health as Biodefense Category B agents.

An estimated 2 million infectious disease-linked cancer cases occurred worldwide in 2008, many attributable to intracellular pathogens like Human Papilloma Virus [5]. Infection with the obligate intracellular bacterium *Chlamydia trachomatis* which, like many microsporidia species, replicates in a cytoplasmic parasitophorous vacuole (PV), increases host cellular chromosomal instability in culture [6] and is associated with increased cervical cancer risk *in vivo *[7]. Like other obligate intracellular pathogens, microsporidia exert significant stress on infected host cells. Microsporidia infection alters host cell cycle regulation [8] and can lead to development of multinucleated host cells [9,10]. Multinucleated cells also occur in human tumors and can give rise to lung metastases in nude mice [11]. Microsporidia infection also inhibits p53-associated, caspase 3-mediated apoptosis [12] - an important mechanism by which some viruses promote host cellular transformation. Finally, oxidative stress markers, such as hydrogen peroxide, free radicals and lipid peroxides, are elevated in microsporidium-infected patients [13]. Though reactive oxygen species are generated during beneficial anti-microbial host responses, they also increase both host DNA damage and cancer risk [14]. Thus, we tested the hypothesis that microsporidia infection increases the host cell nuclear mutation rate within infected cultures.

### Approach

The Big Blue™ transgenic mouse (Agilent Technologies, Inc.) measures *in vivo* mutational frequency in response to genotoxic insults and contains ~40 chromosomally-integrated copies of the λ-phage genome as a mutation target [15]. After mutagen exposure, Big Blue™ mouse genomic DNA is packaged into λ phage capsids. Phage that are wild type at the *cII* locus will not plaque at 24°C in G1250 *E. coli*, but mutational inactivation of the *cII* gene allows lytic replication at 24°C. In contrast, both *cII* mutant and *cII*-wild type phage produce plaques at 37°C [16]. Thus, Big Blue™ cell nuclear mutation can be quantified by dividing the number of *cII*-mutant plaques obtained at 24°C by the total number of plaques at 37°C. We obtained heterozygous Big Blue™ murine embryonic fibroblasts (BB-MEFs) by crossing homozygous male Big Blue™ mice with female BALB/c mice. The tissue, minus head and peritoneal cavity contents, from 2–2.5-week-old embryos was minced and trypsinized. Single cell suspensions were cultured in Dulbecco’s modified Eagle’s medium (DMEM) with 10% fetal bovine serum (FBS) and frozen in liquid nitrogen until use.

**Figure 1:**
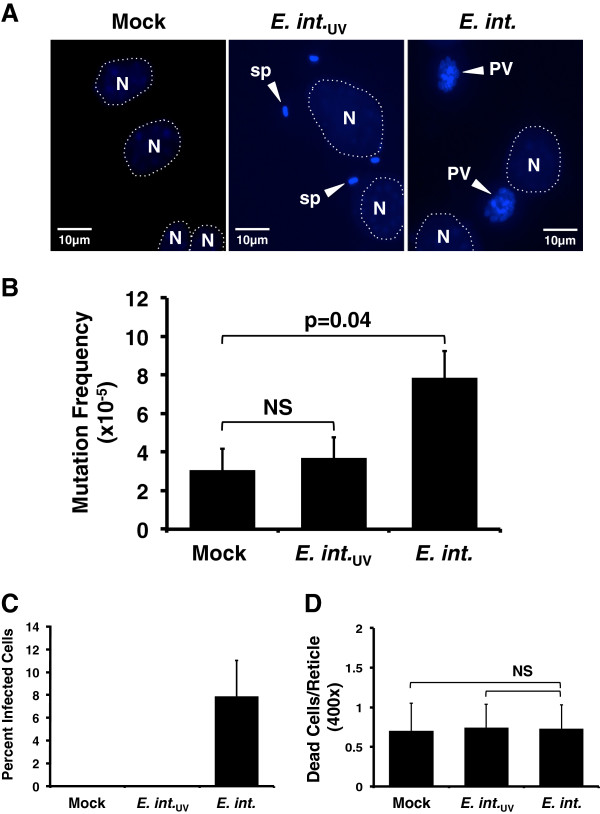
**Productive *E. intestinalis *infection increases host cellular nuclear mutation frequency.** BB-MEF cultures were: i) mock (mock)-infected; ii) *E. intestinalis* (*E. int.*)-infected 5 spores/cell, or iii) infected with ~5 spores/cell of UV-inactivated, *E. intestinalis* (*E. int.*_UV_) refed with DMEM, and incubated at 37°C. Panel **A**. Fluorescent micrographs from mock, *E. intestinalis*- or *E. intestinalis*_UV_-infected BB-MEFs at 42 hpi. Photographs were taken at 630× (oil immersion) using an ultraviolet filter for simultaneous visualization and morphological differentiation of DAPI-stained host cell nuclei and Uvitex-stained spores and parasitophorous vacuoles (PV), all of which appear blue. Because Uvitex-stained spores and PVs fluoresce comparatively more intensely, host cell nuclei are circled to improve visualization. Symbols: N = host cell nucleus; sp = spore; PV = parasitophorous vacuole. Scale bars are 10 microns. Panel **B**. At 42 hpi, 1 x 10^7^ cells were trypsinized, pelleted, quick-frozen, and stored at -80°C. Genomic DNA was purified using the RecoverEase DNA Isolation kit (Agilent Technologies, Inc.). Transpack (Agilent Technologies, Inc.) extracts were used to package genomic λ-phage DNA. Packaged phages were analyzed for *cII* mutation frequency as described. Panel **C**. At 42 hpi, BB-MEF cultures on coverslips were acetone:methanol fixed, stained with 0.01% Uvitex 2B (Polysciences, Inc.) and 1 ug/ml 4’,6-Diamidino-2-Phenylindole, Dichloride (DAPI; Life Technologies, Inc.), and mounted in Vectashield (Vector Laboratories, Inc.). Infectious foci and host cell nuclei were counted in 20 random 630x fields/coverslip using an Olympus BH-2 fluorescent microscope. Percentage of infected cells/reticle was then calculated. Panel **D**. At 42 hpi, BB-MEF cultures on coverslips were assayed using the “Live/Dead” cell viability kit (Molecular Probes, Inc.). Average number of dead cells/400x reticle in 20 random fields/group is plotted. In panels B-D, results average 3 replicates in 3 independent experiments. Means were compared with the unpaired Student’s t test using Microsoft Excel; p values < 0.05 = significant. NS = not significant.

Infectious *E. intestinalis* spores were propagated in Vero African Green Monkey kidney cells (ATCC CCL-81) cultured in DMEM with 2% FBS. After 10–12 days in culture, spores were harvested by centrifugation, washed with 0.25% sodium dodecyl sulfate and sterile water, counted and stored as described [17]. *E. intestinalis* spores (*E. intestinalis*_UV_) were inactivated by 1.14 J/cm^2^ of ultraviolet (UV) light in a Spectroline XL-1500 cross linker and did not produce PVs in subsequent infections (Figure 1A).

## Results

To ascertain infected cell mutation frequency, BB-MEFs were mock-infected (exposed to medium without spores), *E. intestinalis*-infected or infected with *E. intestinalis*_UV_ at 37°C for 42 hours (h). In preliminary time course studies, 42 hours post-infection (hpi) was the latest time at which no difference in host cell death between mock- and *E. intestinalis* BB-MEFs was observed. Genomic DNA, purified from replicate cultures, was packaged into λ phage capsids *in vitro*. G1250 *E. coli* suspensions were infected with phage populations, plated in soft agar and incubated either under non-selective (37°C) conditions for 16 h or selective (24°C) conditions for 48 h [18]. All putative mutant plaques were re-plaqued at 24°C before scoring as mutants and the *cII* mutation frequency was determined as described above. Interestingly, the *cII* mutation frequency was increased by 2.5-fold in *E. intestinalis* productively-infected versus mock-infected cells (Figure 1B; 7.84 × 10^-5^+/-1.19 × 10^-5^ versus 3.07 × 10^-5^ +/-1.08 × 10^-5^; p = 0.04). In contrast, the mutation frequency in BB-MEFs exposed to *E. intestinalis*_UV_ was similar to that in mock-infected cells (3.69 × 10^-5^+/-1.08 × 10^-5^ versus 3.07 × 10^-5^ +/-1.08 × 10^-5^; p = 0.70). These data indicate that: i) microsporidia infection increases host nuclear mutation frequency 2–3 fold; and ii) the observed mutation frequency increase depends upon infection with viable spores.

To confirm infection, *E. intestinalis*-infected BB-MEF cells were stained with Uvitex 2B (a chitin-specific, fluorescent stain that labels free microsporidia spores and those within PVs) and DAPI (to label host cell nuclei), and intracellular microsporidia PVs and host nuclei were counted. Approximately 8% infection was achieved in *E. intestinalis*-infected BB-MEFs at 42 hpi (Figure 1C). *E. intestinalis*-infected BB-MEFs contained morphologically-normal PVs (Figure 1A and [19]). In contrast, PVs were absent in mock- and *E. intestinalis*_UV_-infected cells (Figure 1A,C). UV-inactivated spores adhered to BB-MEFs (Figure 1A), suggesting that spore attachment alone does not increase host mutation frequency. To eliminate the possibility that mutation frequency was increased by DNA damage subsequent to pathogen-induced host cell death, dead cells were quantified in all experimental groups. No significant difference was observed in percent dead cells at 42 hpi between the groups (Figure 1D). Furthermore, average host cell nuclei/field was the same in all groups (see Additional file 1: Figure S1). Thus, the host cell nuclear mutation rate increase is not an artifact of host cell death.

## Discussion

These data demonstrate that: i) *E. intestinalis* infection increases host mutation frequency in culture; and ii) this increase requires some post-attachment step(s) in microsporidia development. Because the Big Blue™ assay does not detect mutations: i) in other parts of the phage/host genome; or ii) that do not alter *cII* gene function, these data likely underestimate MEF nuclear mutation frequency. However, this assay determines whether mutation frequency changes under different experimental conditions – such as infection with various pathogens. For example, gastric infection with the bacterium *Helicobacter pylori* and liver infection with the fluke *Fasciola hepatica* increase Big Blue™ mouse mutation frequencies 5-fold and 2-fold *in vivo*, respectively [18,20]. Although we cannot determine absolute mutation frequency in microsporidia-infected cells, the data clearly demonstrate that microsporidia infection is genotoxic. The magnitude by which *E. intestinalis* increases mutation frequency is within the range observed for other pathogens [18,20] and the carcinogen N-ethyl-N-nitrosourea [21]. It is likely, however, that a higher mutation frequency would be observed if infection efficiencies higher than 8% (Figure 1C) could be obtained without reducing host cell viability.

Microsporidia infection could increase host cellular mutation frequency by several mechanisms. First, microsporidia-infected patients have elevated levels of reactive chemical species [13], suggesting that microsporidia-induced, oxidative DNA damage may elevate host DNA mutation frequency. Second, microsporidia could secrete effectors that directly damage host DNA or indirectly increase mutation frequency by interfering with host DNA repair. For example, *H. hepaticus* and other Gram-negative pathogens secrete cytolethal distending toxins that damage host DNA and increase host cell genomic instability [22]. Notably, microsporidia infection interferes with p53-induced apoptosis [12], indicating that this pathogen may secrete effectors that manipulate pathways linking DNA repair and apoptosis. In either case, mutation frequency in uninfected “bystander” cells may increase, promoting tumor formation even if infected cells do not survive. Finally, the *E. intestinalis* PV may interfere with host DNA segregation, as does that of *C. trachomatis*[6]. As the Big Blue™ assay can neither determine the specific DNA damage mechanism nor discriminate between infected cells and non-infected bystander cells, a more detailed evaluation of the mechanism by which microsporidia-infection elevates host mutation frequency is necessary.

Microsporidia can establish chronic infections [4], even after anti-fungal therapy [3]. Thus, long-term infection has the potential to significantly alter host genomic stability within affected organs. Microsporidia also infect chronically immunosuppressed individuals, who have increased cancer incidence [23]. Though microsporidiosis is observed in cancer patients [24,25], it is assumed to be a byproduct of immunosuppression. The possibility that microsporidia infection contributes to cancer induction or progression has not, therefore, been seriously considered. Our data indicate a possible connection between microsporidiosis and cancer induction that should be further explored. Though the immune response helps control microsporidia infection, inflammation-derived reactive oxygen species can damage DNA. Furthermore, growth factors and cytokines associated with inflammation may support cellular proliferation, migration and metastasis, contributing to cancer development independent of DNA damage. Although microsporidia infection is unlikely to be a proximal cause for cancer, it is plausible that chronic infection, in combination with other genotoxic insults, would increase cancer risk in individuals who contract microsporidiosis, even if infection is asymptomatic.

## Abbreviations

E. intestinalis: *Encephalitozoon intestinalis*; AIDS: Acquired immunodeficiency syndrome; HIV: Human immunodeficiency virus; PV: Parasitophorous vacuole; BB-MEF: Big Blue™ embryonic fibroblast; DMEM: Dulbecco’s modified Eagle’s medium; FBS: Fetal bovine serum; UV: Ultraviolet; E. intestinalisUV: UV-inactivated *E. intestinalis* spores; h: Hours; hpi: Hours post-infection; H. hepaticus: *Helicobacter hepaticus*; C. trachomatis: *Chlamydia trachomatis*; DAPI: 4’, 6-Diamidino-2-Phenylindole, Dichloride.

## Competing interests

The authors declare that they have no competing interests.

## Authors’ contributions

CL participated in the experimental design and performed microsporidia infections, percent infectivity and cell death assays, statistical analyses, drafted the figures, and assisted in manuscript preparation. MS performed animal husbandry tasks, BB-MEF isolation/culture, and quantified mutation frequencies. RVS designed the overall study, performed data analysis/trouble shooting, drafted part of the manuscript, and provided critical reagents. JRH designed experiments, analyzed data, drafted part of the manuscript, reviewed the manuscript and figures, and provided critical reagents. All authors reviewed the manuscript before submission. All authors read and approved the final manuscript.

## Supplementary Material

Additional file 1: Figure S1 Microsporidia infection does not induce host cell loss at 42 hpi. Host nuclei counts were performed on: i) replicate mock (mock)-infected; ii) *E. intestinalis* (*E. int.*)-infected; or iii) UV inactivated, *E. intestinalis* (*E. int.*_UV_)-infected BB-MEF monolayers. At 42 hpi, BB-MEF cultures on glass coverslips were fixed with 1:1 acetone: methanol and DAPI stained. Stained cells were mounted in Vectashield (Vector Laboratories, Inc.) and the number of host cell nuclei were counted in 20 random 630x fields per coverslip using an Olympus BH-2 fluorescent microscope. Host nuclei/reticle was then calculated for each group and plotted. The results shown are averaged from 3 replicates in 3 independent experiments. Means were compared with the unpaired Student’s t test using Microsoft Excel; p values below 0.05 were considered significant. NS = not significant. Click here for file
