# Supplementary figures and images for: Encephalitozoon intestinalis infection increases host cell mutation frequency
Source: Infect Agent Cancer. 2013 Nov 4;8:43. doi: 10.1186/1750-9378-8-43 (PMC4174903; doi:10.1186/1750-9378-8-43)

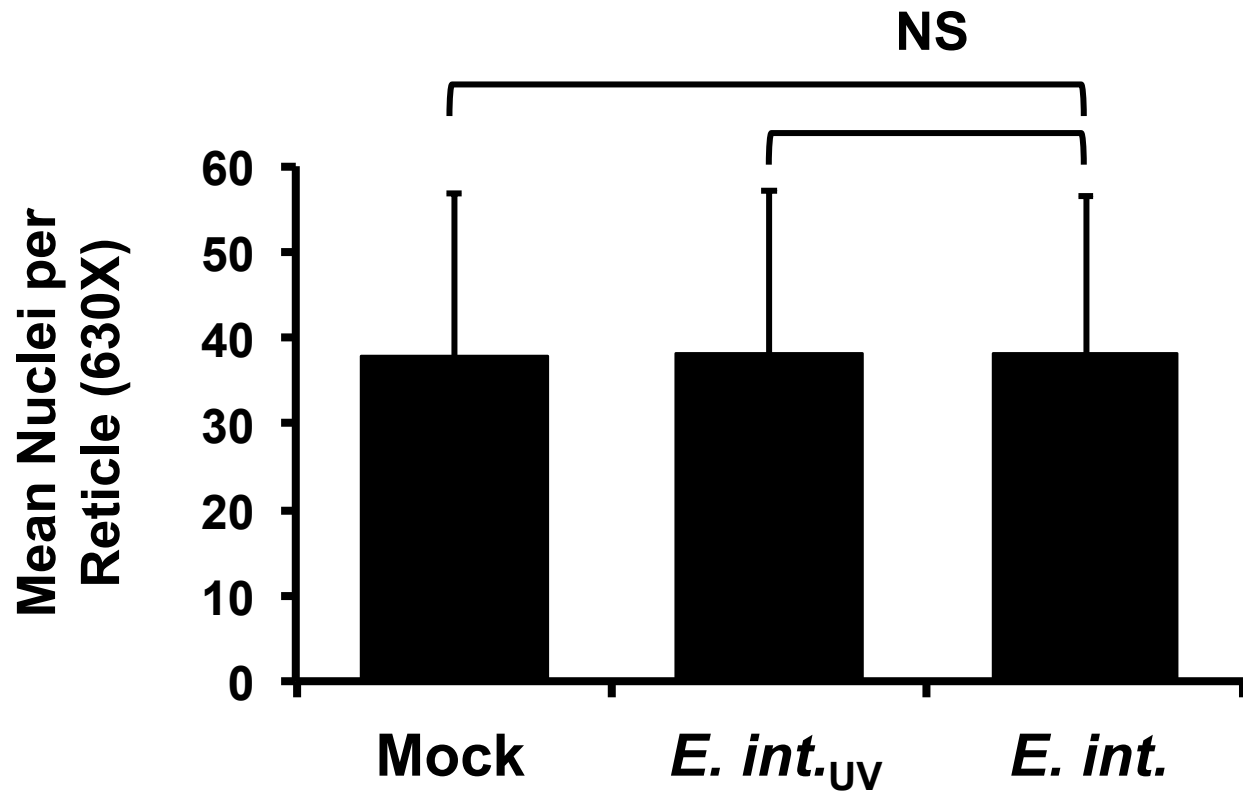

Supplement: Additional file 1: Figure S1 — Microsporidia infection does not induce host cell loss at 42 hpi. Host nuclei counts were performed on: i) replicate mock (mock)-infected; ii) E. intestinalis (E. int.)-infected; or iii) UV inactivated, E. intestinalis (E. int.UV)-infected BB-MEF monolayers. At 42 hpi, BB-MEF cultures on glass coverslips were fixed with 1:1 acetone: methanol and DAPI stained. Stained cells were mounted in Vectashield (Vector Laboratories, Inc.) and the number of host cell nuclei were counted in 20 random 630x fields per coverslip using an Olympus BH-2 fluorescent microscope. Host nuclei/reticle was then calculated for each group and plotted. The results shown are averaged from 3 replicates in 3 independent experiments. Means were compared with the unpaired Student’s t test using Microsoft Excel; p values below 0.05 were considered significant. NS = not significant. [file 1750-9378-8-43-S1.pdf]
